# Progressing Regenerative Medicine: Integrating Bioprinting Platforms for Stem Cell Applications

**DOI:** 10.1155/sci/3319102

**Published:** 2026-07-27

**Authors:** Resmi Rajalekshmi, Devendra K. Agrawal

**Affiliations:** ^1^ Department of Translational Research, College of the Osteopathic Medicine of the Pacific, Western University of Health Sciences, Pomona, California, USA, westernu.edu

**Keywords:** 3D bioprinting, cell viability, computer-aided design, healthcare, regenerative medicine, stem cells

## Abstract

Advancements in regenerative medicine are increasingly driven by the convergence of stem cell biology and bioprinting technologies. Stem cells have the potential to be used to engineer functional tissues and organs because of their innate ability to self‐renew and differentiate. But conventional methods that use xenografts and decellularized scaffolds have issues with biocompatibility and architectural complexity. This review critically evaluates bioprinting strategies tailored for stem cell applications, including the selection of stem cell sources, bioink formulation, crosslinking methodologies, and dynamic culture systems. Particular focus is given to ensuring cell viability, lineage‐specific differentiation, and functional integration post‐fabrication. The review also highlights recent advancements in printing complex tissues such as skin, neural, cardiac, hepatic, and musculoskeletal constructs using embryonic, adult, and induced pluripotent stem cells (iPSCs). Current challenges, ranging from bioink standardization to vascularization, are addressed alongside emerging innovations like microfluidic printing and gene‐activated matrices. By evaluating the integration of bioprinting with stem cell technologies, this article underscores their synergistic potential to address critical gaps in tissue engineering and lays the foundation for translational applications in clinical and pharmaceutical domains.

**Summary**

• Stem bioprinting enables biomimetic tissue engineering.
• Bioprinting regulates spatial cell organization and microenvironments.
• Bioinks replicate the extracellular matrix for stem cell functionality.
• Vascularization and scalability hinder clinical translation.
• Advanced bioprinting strategies improve tissue complexity.

## 1. Introduction

Regenerative medicine is an emerging interdisciplinary field focused on repairing, replacing, or regenerating damaged tissues and organs through the integration of biological and engineering approaches. A central component of this field is the use of stem cells, which possess the capacity for self‐renewal and multilineage differentiation, enabling the generation of specialized cell types required for tissue homeostasis and repair. These properties have facilitated the development of in vitro models for disease investigation, drug screening, and tissue engineering applications [1, 2]. Recent advances have further demonstrated the potential of stem cell‐based strategies for organogenesis and functional tissue regeneration [3, 4].

Conventional tissue engineering approaches, including xenografts and decellularized scaffolds, have achieved partial success but remain limited by issues such as immunogenicity, donor tissue scarcity, and inadequate replication of the native tissue microarchitecture [5, 6]. These limitations highlight the need for advanced fabrication techniques capable of reproducing the spatial organization, cellular heterogeneity, and extracellular matrix composition of native tissues. In this context, three‐dimensional (3D) bioprinting has emerged as a promising platform for the precise fabrication of biomimetic constructs.

3D bioprinting is an additive manufacturing technique that enables the layer‐by‐layer deposition of bioinks composed of living cells, biomaterials, and bioactive factors [7]. Compared with conventional scaffold‐based methods, bioprinting offers greater control over spatial resolution and structural complexity, enabling the generation of tissue constructs with a defined architecture and composition [8]. This level of control is particularly advantageous for stem cell applications as it facilitates the regulation of cell distribution, microenvironmental cues, and cell–matrix interactions that govern cellular behavior, including proliferation, differentiation, and maturation.

The integration of stem cell biology with bioprinting technologies has significantly advanced tissue engineering strategies. Various stem cell types, including embryonic stem cells (ESCs), adult stem cells, and induced pluripotent stem cells (iPSCs), have been used as cell sources due to their capacity to generate patient‐specific, functionally relevant tissues [9, 10]. When combined with optimized bioinks, growth factors, and controlled culture systems, these stem‐cell‐laden constructs can recapitulate key physiological features of native tissues, thereby enhancing their translational potential.

Despite these advancements, several technical and biological challenges remain. These include the standardization of bioink formulations, the maintenance of cell viability during printing, the vascularization of large‐scale constructs, and the scalability for clinical applications. In addition, regulatory considerations and quality control measures must be addressed to facilitate clinical translation. Emerging strategies, such as microfluidic‐assisted bioprinting, gene‐activated matrices, and advanced biomaterial design, are being investigated to overcome these limitations and improve functional tissue integration.

This review critically examines the current developments in bioprinting platforms for stem cell applications. It focuses on key aspects, including stem cell sources, bioink design, printing technologies, and post‐fabrication culture systems. Furthermore, it discusses existing challenges and future directions, emphasizing the potential of integrated bioprinting and stem cell technologies to advance regenerative medicine and improve therapeutic outcomes.

## 2. Fundamentals of Bioprinting

3D printing uses computer‐aided design (CAD) and segmentation software to convert sequential two‐dimensional (2D) medical images, typically derived from CT or MRI scans, into 3D models [11]. These models can then be printed into physical structures. This technology has been applied across various medical specialties for purposes such as surgical planning, educational modeling, and the creation of implantable medical devices. Traditional 3D printing utilizes powders or gels to fabricate the printed object [12]. However, bioprinting is the automated layer‐by‐layer additive fabrication of structures using living cells and biomaterials, guided by a digital model [13]. By sequentially layering biological materials, bioprinting can create complex, 3D structures with intricate architectures. This method enables the precise deposition of various biomaterials and cell types, facilitating the generation of complex tissue‐like constructs from the ground up.

The progression of bioprinting has shifted from a sequential process of printing scaffolds, followed by cell seeding, to a simultaneous procedure that fabricates the 3D‐bioprinted matrix while incorporating cells. Upon implantation, these cell‐laden structures have the potential to integrate with natural tissues, restoring their function [14].

In bioprinting, CAD software is used to precisely layer cells to replicate the patient’s ECM, allowing for high precision and customization. This technology opens up new possibilities in tissue engineering and regenerative medicine (TERM) by converting 3D imaging data into 3D models embedded with living cells and active biomaterials [15]. Traditional tissue engineering methods have shown success but also have limitations, including inaccurate scaffold fabrication, restricted biomaterial delivery, and unreliable cell delivery, which can lead to improper interactions among cell lines during in vivo implantation [16, 17].

Bioprinting offers significant advantages over traditional tissue engineering methods, including improved automation, precision, and customization [18]. Unlike traditional scaffolds, bioprinting creates microstructures with greater anatomical accuracy, allowing for precise co‐deposition of cells and biomaterials [8]. This technique enables the creation of complex biomimetic tissue systems based on medical imaging, providing greater control over the placement of cells and biomaterials [19]. As a result, it facilitates the customization of key anatomical features essential for neovascularization, perfusion, and cellular communication [20, 21].

**Figure 1:**
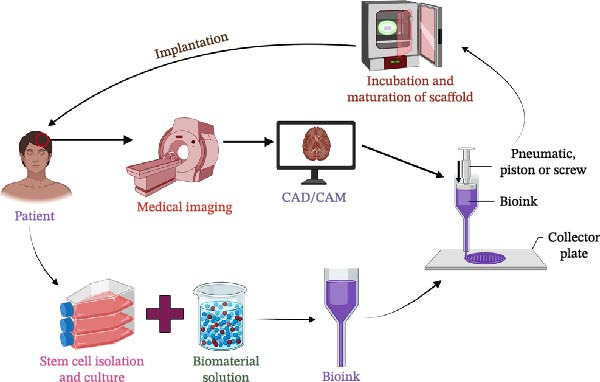
The primary steps in the 3D bioprinting process. CAD, computer‐aided design, CAM, computer‐aided manufacturing.

The 3D bioprinting process encompasses four primary steps, as shown in Figure 1. First, data acquisition involves obtaining 3D models using techniques such as X‐ray, computed tomography (CT), and magnetic resonance imaging (MRI) to scan and reconstruct anatomical structures. Alternatively, models can be created directly using CAD software. These 3D models are then segmented into 2D horizontal slices with customizable size and orientation using specialized software, and the slices are further processed into particles or filaments depending on the bioprinting approach. Second, material selection is critical and involves choosing suitable materials, including cells, growth factors, and hydrogels, based on the requirements of the printed structures and the chosen bioprinting method. These biomaterials, collectively known as bioinks, must meet the printability and mechanical property requirements of the chosen printing method and their biological compatibility are discussed in detail in the bioink formulation section below. Finally, functionalization aims to stimulate the dispersed cells through physical and chemical means to form connections and develop functionalities similar to those of natural tissues or organs.

Bioinks are cell‐compatible formulations composed of living cells, biomaterials, and, when required, bioactive factors that can be deposited by a bioprinter to generate organized tissue constructs. In the context of stem cell bioprinting, bioinks must provide both a printable matrix and a biologically supportive microenvironment. Detailed considerations regarding bioink composition, biological compatibility, and printer‐specific rheological requirements will be discussed in the upcoming sections.

## 3. Types of Bioprinting Technologies

**Figure 2:**
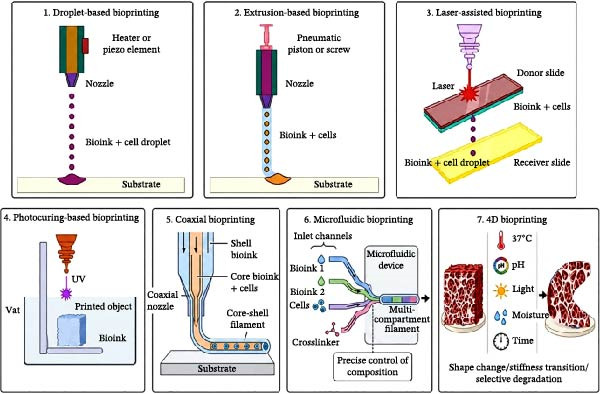
Schematic diagram showing different types of bioprinting methods.

A variety of bioprinting processes, including droplet‐, extrusion‐, laser‐, and photocuring‐based bioprinting, as well as coaxial, microfluidic, and 4D bioprinting, can be employed to achieve the desired goals in additive manufacturing and tissue fabrication (Figure 2).

### 3.1. Droplet‐Based Bioprinting

Droplet‐based bioprinting encompasses various methodologies, including inkjet, acoustic droplet ejection (acoustic), and microvalve bioprinting [22]. Inkjet bioprinting can be further classified into continuous‐inkjet (CIJ), drop‐on‐demand (DOD), and electrohydrodynamic (EHD) jetting [23].

Inkjet bioprinting manipulates bioink solutions through gravity, atmospheric pressure, and fluid mechanics to create droplets. CIJ bioprinting pressurizes the bioink through a nozzle, causing it to break into droplets via Rayleigh‐Plateau instability. DOD bioprinting, favored for tissue bioprinting, produces droplets on demand using thermal, piezoelectric, or electrostatic actuators [24]. DOD inkjet bioprinters use three mechanisms to generate droplets: thermal inkjet (TIJ), piezoelectric inkjet (PIJ), and electrostatic bioprinting [23]. The TIJ uses a thermal actuator, while PIJ printers use a piezoelectric actuator to heat the bioink and eject droplets. Electrostatic bioprinting ejects droplets using an electrostatic force [25]. EHD jet bioprinting conveys the bioink through a nozzle with an electric field, making it suitable for small orifice diameters and high‐concentration bioinks [26]. It forms droplets by creating a Taylor cone at the nozzle tip under high voltages, with droplet size and cell viability influenced by voltage and bioink properties. Key limitations include voltage sensitivity, which requires tight process control, modest throughput compared with extrusion, and a narrower material window [27]. Recent advances in multi‐nozzle EHD systems and real‐time voltage feedback have begun to address the throughput constraint, and hybrid platforms combining EHD with extrusion have been developed to control the resolution of EHD alongside the scalability of extrusion in a single construct [28–30].

Acoustic bioprinting uses gentle acoustic fields to expel droplets from an open pool, circumventing stressors such as heat and high pressure. It involves piezoelectric substrates and interdigitated gold rings to generate surface acoustic waves that exert forces exceeding surface tension, thereby ejecting droplets. This method is sensitive to disturbances from moving printheads and less effective with viscous bioinks [31].

Micro‐valve bioprinting employs electromechanical valves to generate droplets, with back pressure and valve‐gating time dictating the droplet generation mode. This technique is suitable for various proteins and cells, with parameters such as pneumatic pressure and nozzle geometry affecting the droplet volume and cell viability [32]. Micro‐valve bioprinters require lower pneumatic pressure, thereby reducing cell damage, but produce larger droplets and lower resolution than TIJ and PIJ bioprinters [23].

### 3.2. Extrusion Bioprinting

Extrusion‐based 3D bioprinting (EBB) is a widely utilized method that employs pneumatic or mechanically driven dispensing systems to print hydrogels of varying viscosities, enabling the creation of large‐scale models with high cell densities [33]. Its popularity stems from its capability to accommodate a wide range of viscosities and achieve high extrusion pressures. However, it is constrained by limited printing accuracy and by potential adverse effects on cell viability from shear stresses and high pressures.

EBB excels in precisely depositing dense cells, facilitating the development of intricate structures. Despite exhibiting lower cell viability than other bioprinting methods, EBB has proven successful in fabricating diverse tissue constructs, including ear‐shaped scaffolds comprising auricular cartilage and fat, cartilage scaffolds, and hierarchical, cell‐laden structures that simulate multicellular tissues [34, 35].

Parameters such as extrusion pressure and nozzle size significantly impact cell viability, underscoring the importance of meticulous parameter selection for the successful fabrication of 3D tissue constructs. Innovations such as stimuli‐responsive conductive nanocomposite hydrogels have been developed by researchers, showcasing properties such as electrical conductivity, self‐healing, and responsiveness to light and temperature, thereby enhancing the capabilities of the EBB [36]. Continuous technological and material advancements are enhancing its capabilities, positioning it as a promising approach to creating functional, viable 3D tissue constructs for diverse medical applications.

### 3.3. Laser‐Assisted Bioprinting (LAB)

LAB is an advanced printing technology that uses lasers to precisely deposit biomaterials onto substrates without the need for nozzles. This technology includes various techniques such as Laser Guidance Direct Writing (LGDW), Laser‐Induced Forward Transfer (LIFT), Absorbing Film‐Assisted LIFT (AFA‐LIFT), Biological Laser Processing (BioLP), and Matrix‐Assisted Pulsed Laser Evaporation Direct Writing (MAPLE‐DW) [37].

LGDW uses laser‐induced optical forces to pattern living cells in 2D. It employs a weakly focused laser beam directed at a cell suspension to trap and guide cells onto a substrate through gradient forces created by the interaction between the cells and light [38]. However, limited material compatibility has restricted the widespread application of LGDW.

Initially used to transfer metals, LIFT is now employed to deposit various biomaterials, including polypeptides, DNA, and cells. A typical LIFT setup consists of a pulsed laser source, a ribbon structure coated with a bioink, and a receiving substrate [39]. The pulsed laser targets the absorbing layer, causing local evaporation and the formation of high‐pressure bubbles, thereby propelling the cell‐containing material towards the receiving substrate [40]. The precise control of laser parameters is essential due to phenomena such as ablation and plasma generation.

Optimized LIFT variants, including AFA‐LIFT, BioLP, and MAPLE‐DW, have been developed for different applications. AFA‐LIFT features a thicker absorbing layer than traditional LIFT [41]. BioLP facilitates high‐speed, high‐reproducibility mapping of biological materials, incorporating a thick absorption layer and a movable receiving platform with a CCD camera for cell localization [42]. MAPLE‐DW uses a low‐power pulsed laser and a matrix‐embedded transferring layer to enhance laser absorption and energy transfer [43].

The primary advantage of LAB lies in its ability to print biomaterial patterns at high speed and precision on a micron scale. Integrating CAD/CAM systems further enhances the accuracy, enabling near‐single‐cell precision. This capability allows the LAB to create intricate cell arrays that simulate the anisotropy and complexity of tissues [44, 45]. LAB can precisely bioprint various tissue components and reproduce their spatial organization, thereby creating natural tissues and organs with an accurate structure and function.

As a non‐contact, nozzle‐free technology, LAB avoids common issues such as nozzle clogging, non‐reproducibility due to solution viscosity, cross‐infection, and substrate damage [46]. LAB produces smaller droplets with higher precision than inkjet bioprinting. It also allows for higher cell concentrations in the bioink, reducing maturation time. Studies have demonstrated LAB’s effectiveness in constructing multi‐layer cell structures and its suitability for in situ and in vivo bioprinting [47].

Despite these advantages, LAB faces challenges, including limited scalability and potential cell damage from laser heat [48]. Constructing the bioink ribbon is also time‐consuming and complex, particularly for larger structures [49]. Nonetheless, LAB holds significant promise for creating functional and viable 3D tissue constructs for various biomedical applications. Ongoing advancements in technology and materials continue to enhance its capabilities, paving the way for its potential clinical translation.

### 3.4. Photocuring‐Based Bioprinting

Photocuring‐based bioprinting is an innovative tissue engineering methodology that utilizes the photopolymerization properties of photosensitive polymers under controlled lighting conditions to achieve precise, high‐resolution, and high‐speed bioprinting. This cutting‐edge approach addresses common challenges in traditional bioprinting methods, such as nozzle plugging and shear‐stress‐induced effects on cell viability. The two primary techniques in photocuring‐based bioprinting are stereolithography (SLA) and digital light processing (DLP), each distinguished by its unique light‐scanning mode [50].

SLA involves the sequential curing of a light‐sensitive material layer by layer using ultraviolet light [51]. Initially utilized for precise tissue scaffold fabrication with controllable geometries and porous structures, SLA has evolved over the years. Various parameters, such as laser power, scanning speed, exposure time, laser spot size, and wavelength, play pivotal roles in determining the precision achievable with SLA [52].

DLP represents a departure from SLA by enabling the simultaneous solidification of an entire layer using a dynamic mask, significantly enhancing both printing speed and structural integrity [53]. The ability of the DLP technology to fabricate intricate 3D structures with enhanced mechanical properties positions it as a superior method for complex bioprinting applications.

The applications of photocuring‐based bioprinting span various biomedical domains, showcasing its versatility and potential to advance regenerative medicine. Photocuring‐based bioprinting represents a transformative approach to tissue engineering, offering unparalleled precision, speed, and versatility across a myriad of biomedical applications. As research continues to unravel its full potential, this technology holds immense promise for realizing the next generation of functional tissue constructs and advancing toward widespread clinical translation.

### 3.5. Microfluidic and Coaxial Bioprinting

Microfluidic and coaxial bioprinting extend extrusion‐based approaches by integrating concentric needle assemblies or chip‐based flow‐focusing geometries to produce core–shell filaments in a single deposition step. In coaxial configurations, an inner bioink containing cells or a sacrificial porogen is surrounded by a rapidly crosslinking shell, enabling the fabrication of hollow, perfusable channels that function as vascular analogs within the construct [54]. This architecture directly addresses the oxygen and nutrient diffusion limitations that restrict most bulk hydrogel constructs to thicknesses below 200 μm [55, 56]. Stem cells housed in the core compartment benefit from the shell’s immediate structural support, with post‐print viabilities routinely exceeding 85% when shell crosslinking is completed within seconds of deposition. The modality also supports spatial gradients in growth factors or matrix composition along the filament axis, making it well suited to osteochondral and vascular tissue engineering, where distinct zonal properties are required [57].

A key advantage of microfluidic bioprinting is the ability to produce droplet‐in‐shell and multi‐compartment filaments by controlling flow rates in each concentric channel independently [58, 59]. This enables the on‐the‐fly modulation of encapsulated cell density, bioink composition, and crosslink density within a single printed strand. Integration with organ‐on‐chip platforms further extends the utility of this approach for constructing perfusable tissue models suitable for drug screening and disease modeling [60]. Limitations include added hardware complexity, chip or nozzle fouling under prolonged printing, and the need for precise flow‐rate calibration for each new bioink formulation [61].

### 3.6. 4D Bioprinting

4D bioprinting incorporates stimuli‐responsive materials into the bioink, enabling a printed construct to undergo programed shape change, stiffness transition, or selective degradation after fabrication in response to temperature, pH, light, moisture, or cell‐generated mechanical forces [62]. The fourth dimension is time: the construct evolves from its as‐printed geometry to a target configuration that more closely matches the anatomy or mechanical environment of the implant site [63, 64].

Materials employed include shape‐memory polymers, thermoresponsive hydrogels such as poly(N‐isopropylacrylamide) (PNIPAM), and dynamic covalent networks with reversible crosslinks [65, 66]. For stem cell applications, 4D bioprinting offers two practical advantages over static constructs. First, shape morphing triggered after cell loading enables the fabrication of geometries, tubular vascular grafts, and auricular cartilage with a native curvature that cannot be printed directly without mechanical damage to encapsulated cells [67, 68]. Second, programed stiffness progression can replicate the mechanical evolution of the native stem cell niche during tissue development, maintaining cells within the lineage‐promoting stiffness window as the scaffold matures and degrades [69, 70]. Standardization of stimulus protocols, validation of smart material biocompatibility, and scale‐up of shape‐change fidelity remain the principal challenges to clinical adoption [71, 72].

Table 1 compares the principal bioprinting modalities across parameters directly relevant to the stem cell construct design. The trade‐offs summarized in Table 1 should guide modality selection based on the target tissue, the required construct size, acceptable cell loss, and the available infrastructure. Microfluidic bioprinting, a variant of extrusion, additionally enables real‐time concentration gradients of cells and bioactive factors, making it particularly suited to stratified constructs such as the osteochondral tissue.

**Table 1:** Comparison of bioprinting modalities across key parameters relevant to stem cell construct design.

Modality	Typical resolution	Post‐print viability	Viscosity range	Typical cell density	Scalability	Speed	Key advantages/limitations	Typical capital cost
Droplet‐based/inkjet	50–300 μm	80%–95%	1–20 mPa·s	≤10^6^ cells/mL	Low‐moderate	High	1. High speed; low material waste 2. Patterned deposition; single‐cell precision achievable 3. Low‐viscosity inks only; nozzle clogging risk 4. Low cell density; droplet satellite formation	$20k–$120k
Extrusion	100–1000 μm	70%–90%	30 mPa·s‐6 × 10^7^ mPa·s	≤10^8^ cells/mL	High	Moderate	1. Widest viscosity and material range 2. Highest cell density; most scalable 3. Shear stress reduces viability 4. Coarser resolution (>100 μm)	$10k–$200k
Laser‐assisted/LIFT	10–100 μm	>95%	1–300 mPa·s	≤10^8^ cells/mL	Low	Low‐moderate	1. Nozzle‐free; highest cell viability (>95%) 2. High precision; no clogging 3. Slow; low throughput 4. Ribbon preparation complex; high capital cost	$150k–$600k+
SLA/DLP	10–100 μm	60%–95%	Low‐moderate, photocurable	10^6^–10^8^ cells/mL	Moderate‐high	High (DLP), moderate (SLA)	1. Highest geometric fidelity 2. Fast layer curing (DLP); complex architectures 3. Restricted to photoreactive inks 4. Photoinitiator cytotoxicity; light attenuation in thick constructs	$25k–$200k
Microfluidic/coaxial	50–300 μm	70%–95%	1–10^4^ mPa·s	≤10^8^ cells/mL	Moderate	Moderate	1. Perfusable core–shell filaments; vascular analogs 2. In‐line concentration gradients 3. Chip fouling; complex flow‐rate calibration 4. Added hardware; limited scalability	$80k–$300k
4D bioprinting	10–300 μm (base modality‐dependent)	60%–95% (base modality‐dependent)	Modality‐dependent, smart‐material components typically 10–10^4^ mPa·s	10^6^–10^8^ cells/mL	Low‐moderate	Post‐print shape change delayed by minutes to hours	1. Time‐programed morphing; deployable geometries 2. Dynamic stiffness matching to stem cell niche 3. Smart‐material biocompatibility concerns 4. Validation standards and scale‐up immature	Base cost + $10k–$80k premium

*Note*: Values represent ranges compiled from representative published studies; see Sections 3.1–3.6 for primary citations. Capital cost ranges are approximate and exclude maintenance, consumables, and facility costs.

## 4. Stem Cells in Regenerative Medicine

Tissues are complex structures with specific functions formed from cells that share common embryonic origins and morphological features. The field of TERM focuses on repairing damaged tissues by fully restoring or replacing them. Stem cells, which have the remarkable ability to self‐renew and differentiate into various cell types, play a crucial role in supporting TERM technologies.

Stem cells come in three major classes: ESCs, adult stem cells, and iPSCs [1]. ESCs have unlimited proliferative potential and can differentiate into all cell types in the human body, but their use is limited by ethical concerns and availability issues [73]. Adult stem cells are specific to certain tissues and can regenerate, but they have limited capacity to differentiate into a wide range of tissues compared to ESCs [74]. On the other hand, iPSCs, derived from adult cells, can differentiate into various cell types and are increasingly used in tissue engineering due to their potential to generate patient‐specific models [75].

Stem cells have been used in injectable therapies to treat various diseased or damaged tissues, and their use in TERM and bioprinting continues to advance. By utilizing stem cells to produce functional tissue constructs, researchers aim to effectively repair or model the tissues of interest.

## 5. Integrating Bioprinting With Stem Cells

Stem cell culture, expansion, and differentiation present significant challenges in tissue engineering, highlighting the need for innovative approaches such as bioprinting. Maintaining stem cell viability and functionality in culture is a critical yet complex endeavor due to the delicate balance of growth factors, nutrients, and signaling cues required for their survival and proliferation [76]. Additionally, scalability remains a concern as traditional culture methods often struggle to produce the large numbers of cells required for tissue engineering applications [77]. Furthermore, the precise control of stem cell differentiation into specific cell lineages is intricate and requires precise manipulation of biochemical and mechanical cues within the microenvironment [78].

Bioprinting offers a promising solution to these challenges by enabling the precise deposition of stem cells within 3D scaffolds, allowing for the spatial organization of cells and biomaterials to replicate the native tissue architecture. This technology facilitates the creation of complex tissue constructs with controlled cell density, distribution, and organization, thereby enhancing stem cell survival, proliferation, and differentiation. Furthermore, bioprinting permits the incorporation of growth factors and signaling molecules directly into the scaffold, providing spatiotemporal control over the stem cell microenvironment.

### 5.1. Bioprinting Strategies for Stem Cells

#### 5.1.1. Stem Cell Source

Before bioprinting, selecting the appropriate stem cell source is crucial, tailored to the intended application. ESCs, iPSCs, and adult stem cells, such as mesenchymal stem cells (MSCs), are common options, each with distinct advantages and limitations regarding proliferation capacity, differentiation potential, and ethical considerations.

One significant advantage of stem cells is their theoretically unlimited proliferative capacity, enabling the generation of large numbers of functional cells. Technologies have been developed to induce the differentiation of ESCs and iPSCs into various functional cell types, such as neurons, cardiomyocytes, hepatocytes, β cells, muscle cells, and endothelial cells. Many of these protocols have successfully produced large quantities of functional cells for constructing 3D hepatic, cardiac, and neural tissues [79–83].

As stem cells differentiate, their proliferative capacity decreases, posing a challenge for populating 3D constructs [84]. One approach to overcome this challenge is to guide stem cells towards differentiation into committed or progenitor cells that still retain their proliferative capacity. Once these cells populate the 3D bioprinted structure, further differentiation and maturation can be induced, ultimately enabling the development of functional and mature tissues and organs [85]. Another advantage of pluripotent stem cells in 3D bioprinting is the ability to generate multiple cell types with the same genetic background. This is particularly useful in clinical settings where constructing an artificial organ using cells exclusively sourced from the same individual can mitigate the risk of immune rejection [86]. A cost‐effective and customizable bioprinting platform has been recently developed to create kidney organoids from Nephron progenitor cells (NPCs) derived from iPSCs. This system enables precise cell deposition, allowing for the high‐throughput production of kidney organoids. The organoids express markers of major kidney cell types and can be efficiently generated from as few as 8000 cells [87].

Despite these advancements, safety concerns must be addressed before the clinical application of ESC‐ or iPSC‐based 3D bioprinting technologies. These concerns include the risk of genetic and epigenetic abnormalities and the potential tumorigenicity of undifferentiated pluripotent stem cells. Efforts have been dedicated to enhancing safety by developing ESC and iPSC strains with normal karyotypes, avoiding tumorigenic mutations, and implementing good manufacturing practices (GMP) throughout production to meet clinical application requirements [88]. The emerging clinical applications of ESC‐ and iPSC‐derived cells hold tremendous potential as cell sources for 3D bioprinting of tissues or organs for therapeutic purposes.

In addition to pluripotent stem cells, adult stem cells such as MSCs serve as a versatile and ethically favorable alternative for 3D bioprinting applications. MSCs can be isolated from multiple sources, including bone marrow [89, 90], adipose tissue [91–93], umbilical cord [94, 95], Wharton’s jelly [96], dental pulp [97], synovial fluid [98], and amniotic fluid [99]. MSCs have the potential to differentiate into multiple cell lineages and promote tissue regeneration, making them widely used in the development of the 3D bioprinting technology and tissue repair research [100, 101]. A recent study investigated the use of 3D‐printed SiO2/PTHF/PCL‐diCOOH scaffolds to promote the growth of sheep bone marrow stem cells in vitro. The findings showed that the scaffolds facilitated cell attachment and growth, leading to increased levels of chondrogenic markers and the formation of a cartilage extracellular matrix [102].

#### 5.1.2. Bioink Development

Bioinks serve as the vehicle for delivering stem cells and supporting their growth and differentiation within the printed construct. The design of biomaterial inks must take into account the need for functional matrices that replicate the physicochemical properties of the native state.

First and foremost is biocompatibility, which entails selecting biomaterials that closely mimic the native ECM to support stem cell growth, proliferation, and differentiation without adverse effects. In the realm of bioinks, water‐soluble polymers, commonly denoted as hydrogels, have emerged as the most suitable biological materials. Their structural resemblance to the ECM facilitates the adhesion, proliferation, and differentiation of encapsulated stem cells. Hydrogels employed in 3D bioprinting are primarily classified into two categories: natural and synthetic [103]. Natural hydrogels, including collagen [104], silk [105], alginate [106], fibrin [107], hyaluronic acid (HA) [108], cellulose [109], gelatin [110], and chitosan [111], exhibit high biocompatibility and closely emulate the natural ECM. Conversely, synthetic hydrogels such as polyethylene glycol (PEG) [112], polycaprolactone (PCL) [113], and polylactic acid (PLA) [114] offer customizable mechanical and physical properties. While natural hydrogels are generally more biocompatible, synthetic hydrogels enable greater customization of their mechanical and structural attributes. The decellularized components of the matrix provide a novel bioink source. These components are derived from tissue fragmentation, cell lysis, and isolation of the remaining ECM [115]. Throughout this process, the ECM may compromise its mechanical strength and structural integrity, necessitating a scaffold to preserve the intended form and function during bioprinting.

Another important property is printability, which refers to the ability of the bioink to be extruded, ink‐jetted, or otherwise deposited with high precision to create complex tissue architectures. The rheological properties of the bioink, such as viscosity and shear‐thinning behavior, are critical for enabling the controlled deposition of cells and biomaterials. The molecular mechanisms underlying shear‐thinning and the physicochemical interactions responsible for shape retention differ among bioink classes, and for stem cell bioprinting, bioinks should be specifically designed to exhibit shear‐thinning behavior (defined as a reversible reduction in apparent viscosity under applied shear force, allowing smooth flow through the nozzle during extrusion, followed by viscosity recovery and shape retention after deposition) for smooth extrusion and structural fidelity [116]. A recent study developed bioinks using shear‐thinning and self‐healing hydrogels for bioprinting. Based on silicate nanomaterials and glycosaminoglycan nanoparticles, the hydrogels protected cells during bioprinting. They could create shape‐persistent structures and support cell growth and osteogenic differentiation in murine bone marrow stromal cells, specifically pre‐osteoblasts. In vivo studies in rats confirmed their potential for bone tissue engineering [117].

#### 5.1.3. Microenvironment

Bioink additives are essential in regulating and directing the differentiation of stem cells into specific cell phenotypes or functional characteristics. These additives typically include substances such as calcium phosphate‐based ceramics (e.g., hydroxyapatite (HAP) and β‐tricalcium phosphate (β‐TCP)), bioactive glass, microcarriers, and various bioactive components such as growth factors, cytokines, enzymes, and DNA, which are commonly used for this purpose.

To address the challenge of bone defect reconstruction using 3D bioprinting technology in tissue engineering, a collagen‐based scaffold was fabricated using 3D printing, aided by a gelatin support bath. HAP and bone marrow mesenchymal stem cells (BMSCs) were incorporated to mimic bone composition. The blend of HAP and collagen exhibited suitable rheological properties for 3D printing and enhanced scaffold strength. The gelatin support bath effectively maintained scaffold dimensions with the designed patterns at room temperature. BMSCs within the scaffold remained viable, proliferated, and expressed high levels of alkaline phosphatase. Overall, the printed collagen‐based scaffold demonstrated biocompatibility, mechanical strength, and bioactivity, offering a promising approach for bone tissue engineering via 3D bioprinting [118].

In another study, the focus was on addressing rheological and biological challenges in creating extrudable bioactive hydrogels through the incorporation of Borate glass into alginate‐gelatin hydrogel at various weight‐to‐volume percentages. This addition resulted in enhanced stiffness of the hydrogel. Human adipose‐derived mesenchymal stem cells (hADSCs) were uniformly mixed into the bioink at a concentration of 1 × 10^6^ cells/mL and cultured under both static and dynamic conditions. Increased viability after 7 days with a borate glass content of 0.15% or less suggested the potential utility of highly resorbable calcium‐releasing biomaterials, such as bioactive glasses, in modifying hydrogels suitable for bioprinting cellularized 3D structures [119].

By self‐assembling cells within bioinks, microcarriers provide a favorable environment for enhanced cell interaction and the fabrication of more functional tissue constructs [120]. Literature reported a novel strategy to overcome challenges in fabricating functional grafts of clinically relevant size by combining bioprinting with microcarrier technology. By utilizing MSC‐laden PLA microcarriers encapsulated in gelatin methacrylamide‐gellan gum bioinks, living constructs were fabricated via bioprinting. The incorporation of microcarriers improved the compressive modulus of the hydrogel constructs, enhanced cell adhesion, and supported osteogenic differentiation and bone matrix deposition by MSCs. Additionally, bilayered osteochondral models were successfully fabricated using a microcarrier‐laden bioink for the bone compartment. These findings highlighted the potential of this innovative microcarrier‐based biofabrication approach for creating bone and osteochondral constructs [121].

To improve the alginate bioink’s printability, structural stability, and biological activity, bioinks were formulated using bone morphogenetic protein‐2 (BMP‐2)‐loaded poly(lactic‐co‐glycolic acid) (PLGA) nanoparticles and alginate for MSC printing and osteogenic differentiation. The inclusion of PLGA nanoparticles enhanced the mechanical properties and printability of the bioink. BMP‐2‐loaded PLGA nanoparticles (NPBMP‐2) demonstrated sustained BMP‐2 release for up to 2 weeks. In vitro studies showed that the alginate and NPBMP‐2 bioink significantly induced MSC osteogenesis, evidenced by increased calcium deposition, alkaline phosphatase activity, and the expression of osteogenic markers [122]. Another group of researchers developed a thermoresponsive nanocomposite (TNC) bioink using poly(organophosphazene) loaded with bone morphogenetic protein‐2 (BMP‐2) and transforming growth factor‐beta1 (TGF‐β1) for 3D printing. The TNC bioinks, with shear‐thinning and self‐healing properties, enabled smooth extrusion and favorable printing outcomes. The incorporation of BMP‐2 and TGF‐β1, both crucial for osteogenesis, enhanced the scaffold’s biological activity. Specifically, the TNC bioink loaded with these growth factors exhibited 2.2 times greater cell migration than the one without them [123]. Other growth factors used as additives in bioink include vascular endothelial growth factor (VEGF) [124] and basic fibroblast growth factor (bFGF) [125], which promote the growth and differentiation of BMSCs and hUMSCs.

DNA‐containing inks are biologically active materials used in tissue engineering, facilitating tissue formation and extracellular matrix production via nonviral gene delivery and showing promise in enhancing scaffold functionality. Recently, a study introduced a novel pore‐forming bioink to address the challenge of regenerating complex tissues and organs by enabling controlled gene delivery in bioprinted tissues. This bioink, formed by crosslinking alginate and methylcellulose, displayed increasing porosity over time, thereby enhancing non‐viral gene transfer to stem cells in vitro. The modulation of porosity enabled either rapid, transient transfection or slower, sustained transfection of cells in vivo. These bioinks facilitated the zonal positioning of stem cells and plasmids encoding osteogenic or chondrogenic genes within 3D‐printed thermoplastic fiber networks, resulting in mechanically reinforced, gene‐activated constructs. *In vivo*, the bioprinted tissues developed vascularized bone overlaid with stable cartilage [126].

#### 5.1.4. Crosslinking Methods

Crosslinking is a crucial process that significantly affects the mechanical and physicochemical properties of bioprinted constructs, as well as the behavior of encapsulated living cells. The chosen crosslinking method must be cytocompatible to ensure that it does not compromise cell viability or functionality. Crosslinking processes can be divided into two main categories: physical and chemical methods, distinguished by their respective mechanisms. Chemical methods are favored for their versatility and stability under physiological conditions, making them extensively utilized in various applications. In contrast, physical crosslinking relies on secondary forces such as hydrophobic interactions, hydrogen bonding, and van der Waals forces [127]. The hydrogels, formed through weak interactions, have limited stability and mechanical strength. However, their reversibility is advantageous in tissue engineering, allowing them to dissolve and release encapsulated molecules in response to local pH changes or changes in the body’s microenvironment [128]. In a recent study, researchers introduced an innovative 3D bioprinted hydrogel scaffold formed from a blend of sodium alginate and gelatin, crosslinked using a CaCl_2_ solution. This scaffold, when combined with neural stem cells (NSCs) and oligodendrocytes (OLGs), demonstrated exceptional compatibility for both cell types. Following transplantation into fully transected rat spinal cords at the T8−9 level, the cell‐loaded 3D bioprinting scaffold showcased promising outcomes. Notably, the group treated with cells/scaffold displayed significant improvements in nerve regeneration and motor functional recovery, outperforming both the scaffold‐only and spinal cord injury groups in the study [110].

Chemical cross‐linking reagents such as glutaraldehyde [129], 1‐ethyl‐3‐(3‐dimethylaminopropyl) carbodiimide hydrochloride (EDC) [130], glyceraldehyde [131], and formaldehyde [132] are commonly utilized in the preparation of various hydrogels. However, their applicability to 3D bioprinting with cells is limited by their potential cytotoxicity [133]. In contrast, genipin, a natural chemical crosslinker derived from gardenia fruit, is regarded as the least cytotoxic and thus more suitable for bioinks containing cells [134]. Genipin cross‐links functional amine groups on natural polymers, yielding a more stable gel compared to carbodiimides like EDC due to a longer crosslinking distance [135]. A recent study examined the use of genipin‐crosslinked 3D‐printed gelatin scaffolds for regenerating cartilage in the temporomandibular joint (TMJ). Crosslinking with genipin improved stability and mechanical properties without causing cytotoxicity. hBMSCs demonstrated chondrogenic differentiation on the scaffolds, with a reduced hypertrophic tendency, as confirmed after 21 days. Despite its utility in creating supportive hydrogels for stem cell differentiation, its high cost and limited research have hindered its widespread adoption in cell bioprinting [136].

Protein‐based hydrogel bioinks often leverage enzymatic cross‐linking, an alternative form of chemical cross‐linking. Key enzymes utilized include phosphopantetheinyl transferase (PPTase) [137], mushroom tyrosinase [138], Horseradish peroxidase (HRP) [139], and microbial transglutaminase [140]. The enzymes commonly used in injectable hydrogels and bioinks are typically present in the human body. The enzymatic cross‐linking process is compatible with stem cells and occurs under gentle conditions such as neutral pH and physiological temperature, providing specificity that prevents toxicity and enables control over mechanical properties. A study discussed the use of enzymatic crosslinking to create hybrid hydrogels by combining silk fibroin (SF) with tyramine‐substituted SF (SF‐TA) or gelatin (G‐TA). These hybrid hydrogels demonstrated adjustable gelation kinetics, mechanical properties, and bioactivity. The study found that both SF‐TA and G‐TA improved gelation kinetics, mechanical properties, and enzymatic degradation. Additionally, incorporating cyclic RGD peptide or G‐TA content enhanced the bioactivity of the hydrogels, thereby increasing the metabolic activity of the encapsulated hMSCs [141]. Although they are expensive and challenging to produce, enzymatic hydrogels provide a suitable microenvironment for stem cells and are a significant approach to developing injectable hydrogels and bioinks [142].

Photocrosslinking stands out as a widely adopted chemical crosslinking method in 3D bioprinting due to its high efficiency and precise control over spatial and temporal parameters, which are essential for creating durable tissue constructs [143]. This technique uses photoinitiators such as Irgacure 2959 and photo‐reactive polymers, enabling the formation of covalent bonds by adjusting parameters such as light intensity, exposure duration, and illumination area [144]. These photo‐induced reactions offer rapid cross‐linking, precise control over reaction conditions, maintenance of the hydrogel shape at room temperature, and spatial–temporal regulation. The optic‐fiber‐assisted bioprinting (OAB) process efficiently photo‐crosslinked methacrylated hydrogels, enabling the fabrication of biofunctional structures laden with hADSCs using methacrylated gelatin (GelMA), collagen, and a decellularized extracellular matrix (dECM). This method was recently applied to skeletal muscle regeneration, resulting in significantly higher levels of alignment, myogenic differentiation, and myogenic activity in hADSCs compared to those in conventional crosslinking methods. Furthermore, *in vivo* studies demonstrated that the hASC‐laden construct induced greater muscle regeneration than the cell construct lacking topographical cues [145].

The literature also illustrates how combining these crosslinking techniques can enhance the mechanical properties and cytocompatibility of hydrogel bioinks. In a recent study, SF‐based inks were developed for in situ applications via a covalent crosslinking process that involved pre‐photo‐crosslinking prior to printing and in situ enzymatic crosslinking. Two different molecular weights of SF were characterized, revealing that covalent bonds and shear forces enhanced the transition to β‐sheets, thereby promoting rapid stabilization. These hydrogels demonstrated good mechanical properties, long‐term stability, and resistance to enzymatic degradation over 14 days, maintaining their secondary structure and swelling behavior. They also supported cell viability, with a significant increase in hBMSCs’ metabolic activity from day 1–7 [146].

In another study, researchers discussed using a dual‐crosslinking approach with Alg/Gel/GelMA inks. This approach allowed for the fabrication of stable scaffolds with low cytotoxicity. The method used a low percentage of the lithium phenyl‐2,4,6‐trimethylbenzoylphosphinate (LAP) photoinitiator and physical Ca^2+^‐assisted stitching to achieve extensive stability. The study demonstrated that ink formulation I (1% Alg/4% Gel/5% GelMA w/v) enabled high printing accuracy and the fabrication of high‐resolution constructs suitable for differentiating rat MSC for bone tissue development in vitro [147].

#### 5.1.5. Dynamic Culture Systems

After printing, the constructs were typically cultured in bioreactors under controlled conditions to promote cell proliferation, differentiation, and tissue maturation. Bioreactor systems provide mechanical stimulation, nutrient perfusion, and oxygenation to mimic the physiological environment and enhance tissue development [148, 149]. A recent study introduced fused deposition modeling (FDM) technology for creating 3D‐printed perfusion bioreactors, overcoming current limitations in tissue and organ modeling. Made from PLA, these bioreactors allowed for flexible design and customization. Validated for culturing human mesenchymal stromal cells on collagen scaffolds, they enabled engineered microenvironments of various sizes, confirmed by confocal microscopy. The bioreactors also facilitated interaction between human hematopoietic stem cells and mesenchymal cells, leading to the expansion of the stem cell population. Overall, this approach demonstrated the potential of 3D‐printed perfusion bioreactors to accurately model complex stem cell systems within dynamic 3D microenvironments, offering new avenues for investigating cellular processes [150]. In another study, the efficacy of FDM‐printed bioreactors in sustaining bio‐printed tissue using POx‐Alginate and Cellink Bioink was demonstrated. Murine C2C12 cells were cultured, and hMSCs were induced to differentiate into adipocytes. Over 14 days, the high cell viability suggested adequate nutrient distribution without material‐related issues or contamination. Notably, there were no observable macroscopic changes in tissue size or cell clustering around pores. Histological analysis confirmed the adipogenic differentiation of hMSCs, as evidenced by a multilocular morphology. This successful maintenance and differentiation within the bioreactor system highlighted its dynamic culture environment, facilitating tissue maturation and cell differentiation [151].

#### 5.1.6. Degradation and Remodeling

Cells in a 3D microenvironment require a soft matrix for migration and proliferation. Designing a hydrogel matrix with sustainable degradation is crucial for biofabrication. Dynamic bioink degradation provides space for cell proliferation and regulates microenvironmental cues for stem cells within printed constructs. Various strategies have been reported in the literature to prepare biodegradable bioinks. In a recent study, the oxidation of alginate (OA) was utilized to adjust the degradation rate of alginate‐based bioinks for cartilage tissue engineering. Raw and OA were combined at varying ratios to regulate the degradation rate. These blends were then mixed with gelatin to create degradable bioinks suitable for extrusion bioprinting. The degradation rate was significantly influenced by the OA content, and despite notable mass loss, the printed geometry remained intact throughout a 4‐week in vitro culture period. Moreover, all blends facilitated robust chondrogenic differentiation of MSCs, leading to the development of a hyaline‐like tissue enriched in type II collagen and devoid of calcific deposits [152].

Another group of researchers developed a bioink comprising hMSCs for 3D bioprinting, utilizing HA and alginate as key components. Their investigation extensively characterized this bioink, assessing its physical, mechanical, and biological properties. Notably, the bioink exhibited the properties required for bioprinting applications, including high porosity, swelling capacity, and appropriate rheological behavior. The rate of degradation was found to be temperature‐dependent. Additionally, the bioink exhibited negative surface electrical properties and demonstrated stability under stress conditions. Also, it created an environment supportive of hMSC viability and growth, with high cell viability maintained post‐extrusion [153].

#### 5.1.7. Mechanistic Regulation of Stem Cell Fate in Bioprinted Constructs

A mechanistic understanding of how bioprinted microenvironments govern stem cell behavior is essential for rational construct design. Matrix stiffness is among the most influential biophysical parameters. Soft matrices with elastic moduli in the range of 0.1–1 kPa favor neurogenic differentiation of MSCs, matrices of intermediate stiffness (8 to 17 kPa) promote myogenesis, and stiffer substrates (25 to 40 kPa) drive osteogenesis [154, 155]. These responses are transduced primarily through the Hippo pathway effectors YAP (Yes‐associated protein) and TAZ (transcriptional coactivator with PDZ‐binding motif) [156, 157]. On stiff substrates, YAP and TAZ remain nuclear and transcriptionally active, promoting the expression of osteogenic and myogenic genes [158]. On compliant substrates, cytoplasmic retention of YAP/TAZ shifts the balance toward adipogenic or neurogenic programs [158]. In 3D bioprinted constructs, the bioink’s crosslink density and polymer concentration influence the effective stiffness, which in turn modulates YAP/TAZ localization in an engineered manner [159].

Adhesion ligands embedded in the bioink provide additional lineage cues. RGD peptides engage integrins, activate focal adhesion kinase (FAK), and upregulate Rho/ROCK‐mediated cytoskeletal tension, promoting osteogenic and smooth muscle differentiation via MRTF‐A nuclear translocation [160, 161]. The laminin‐derived peptides IKVAV and YIGSR activate distinct downstream cascades that promote neuronal specification [162]. Adhesion motif selection is therefore an independent design variable from the polymer backbone choice.

Shear stress during extrusion bioprinting activates mechanosensitive Piezo1 channels, induces calcium influx, and transiently phosphorylates ERK1/2 and p38 MAPK [163, 164]. At shear rates above ~5000 s^−1^, MSC viability decreases, and surface marker expression is altered. iPSC‐derived cardiomyocytes are more susceptible than MSCs due to their limited capacity for cytoskeletal remodeling, which is why valve‐based or light‐assisted deposition is preferred for cardiac cell types [165].

Crosslink density also governs intraconstructs oxygen and nutrient gradients. Dense hydrogels restrict diffusion, stabilize HIF‐1α, and promote chondrogenesis through SOX9 upregulation, while more porous architectures support osteogenic and vascular differentiation via mTOR‐dependent pathways [166, 167]. Additionally, matrix degradation products, including HA oligosaccharides and gelatin peptides, signal through CD44 and toll‐like receptors, modulating the inflammatory state and epigenetic programing in encapsulated stem cells [168, 169]. These mechanisms collectively inform the bioink formulation, crosslinking strategy, and modality selection for a given target lineage.

### 5.2. Unifying Design Principles for Bioprinted Stem Cell Constructs

The tissue‐specific sections that follow share four recurring engineering challenges: matching bioink rheology to the printing modality, aligning matrix mechanics with the target stem cell lineage, selecting an appropriate mechanical conditioning regimen, and tuning the scaffold degradation rate to match endogenous matrix deposition.

#### 5.2.1. Bioink Rheology and Printability

Printable bioinks for extrusion‐based systems need viscosities from ~30 mPa·s to 6 × 10^7^ mPa·s [170]. Shear‐thinning behavior, indicated by flow‐behavior index n below 0.5, is essential for reliable filament formation [171]. A finite yield stress is necessary to maintain filament geometry post‐deposition, though excessive yield stress increases nozzle shear and reduces cell viability. Alginate‐gelatin blends are the most widely used systems because polymer concentration and crosslinker dose independently control viscosity and yield stress.

For inkjet systems, the operative range is narrow: viscosities of 1–10 mPa·s and surface tensions of 0.02–0.07 N/m [172]. For SLA and DLP, photopolymerization kinetics govern printability more than viscosity; gelation time must balance layer fidelity against oxygen inhibition [173]. Modality‐specific rheological requirements should be established before biological optimization as a bioink incompatible with the printer will produce non‐reproducible constructs regardless of its biological composition.

#### 5.2.2. Stem Cell‐Matrix Mechanical Matching

The elastic modulus of the crosslinked bioink must correspond to the stiffness range that directs the target lineage. The established thresholds for MSC differentiation are 0.1–1 kPa for neurogenesis, 8–17 kPa for myogenesis, and 25–40 kPa for osteogenesis [174, 175]. In GelMA, increasing methacrylate substitution from 40% to 80% raises the compressive modulus from ~1 kPa to 15 kPa [176, 177]. For cartilage, with a native equilibrium modulus of 0.5–1 MPa, fiber‐reinforced composites—consisting of a stiff PCL framework combined with a soft hydrogel phase—are needed to separate bulk mechanics from cell‐scale matrix stiffness [178]. Stiffness matching must also account for construct remodeling over time: as cells deposit the matrix and the scaffold degrades, the effective modulus shifts, and this trajectory should maintain cells within the lineage‐appropriate stiffness window throughout the culture period.

#### 5.2.3. Mechanical Conditioning

Static culture does not replicate the biomechanical environment of the native tissue. Cardiac constructs require electrical field stimulation (1–2 Hz, 1–5 V/cm) to synchronize cardiomyocyte contraction and promote myofibril alignment and gap junction formation [179]. Bone and cartilage constructs respond to cyclic compressive loading (5%–15% strain, 0.5–1 Hz), thereby activating integrin‐mediated mechanotransduction and upregulating collagen type I and aggrecan synthesis [180]. Hepatic constructs require perfusion through embedded microchannels to sustain the oxygen gradient necessary for zone‐specific metabolic activity [181]. Vascular and skeletal muscle constructs respond to pulsatile fluid flow and uniaxial cyclic strain, respectively [182, 183]. Conditioning should begin 24–72 h post‐print after cells have established initial matrix contacts [184, 185].

## 6. Bioprinted Tissues

A critical appraisal of the literature reveals that the success or failure of bioprinted stem cell constructs depends on how well the bioink mechanical environment, the printing modality, and the stem cell source are matched to one another and to the target tissue. Several apparent contradictions in the field become intelligible once this tripartite relationship is considered. For instance, alginate‐gelatin bioinks consistently support chondrogenesis of MSCs because their moderate stiffness (1–10 kPa) and limited cell adhesion ligands recreate the avascular cartilage niche, whereas the same bioinks impair osteogenesis unless supplemented with HAP or bone morphogenetic protein‐2, which provide the mineral and growth factor cues absent from the base hydrogel. Similarly, extrusion‐based bioprinting yields superior post‐print viability and osteogenic outcomes for MSCs because MSCs tolerate shear forces and respond positively to the mechanical priming; however, iPSC‐derived cardiomyocytes are sensitive to shear‐induced membrane damage and depolarization, making light‐assisted (SLA and DLP) or valve‐based deposition preferable for cardiac constructs. The recurring observation that constructs performing well in short‐term in vitro assays failing in vivo often reflects a mismatch between initial mechanical properties and the degradation rate of the bioink: if the scaffold softens too quickly, stem cells lose the stiffness cues required to maintain their differentiated state. Recognizing these design principles allows the investigator to move from trial‐and‐error selection toward rational construct engineering. The tissue subsections below present selected studies illustrating these principles.

**Figure 3:**
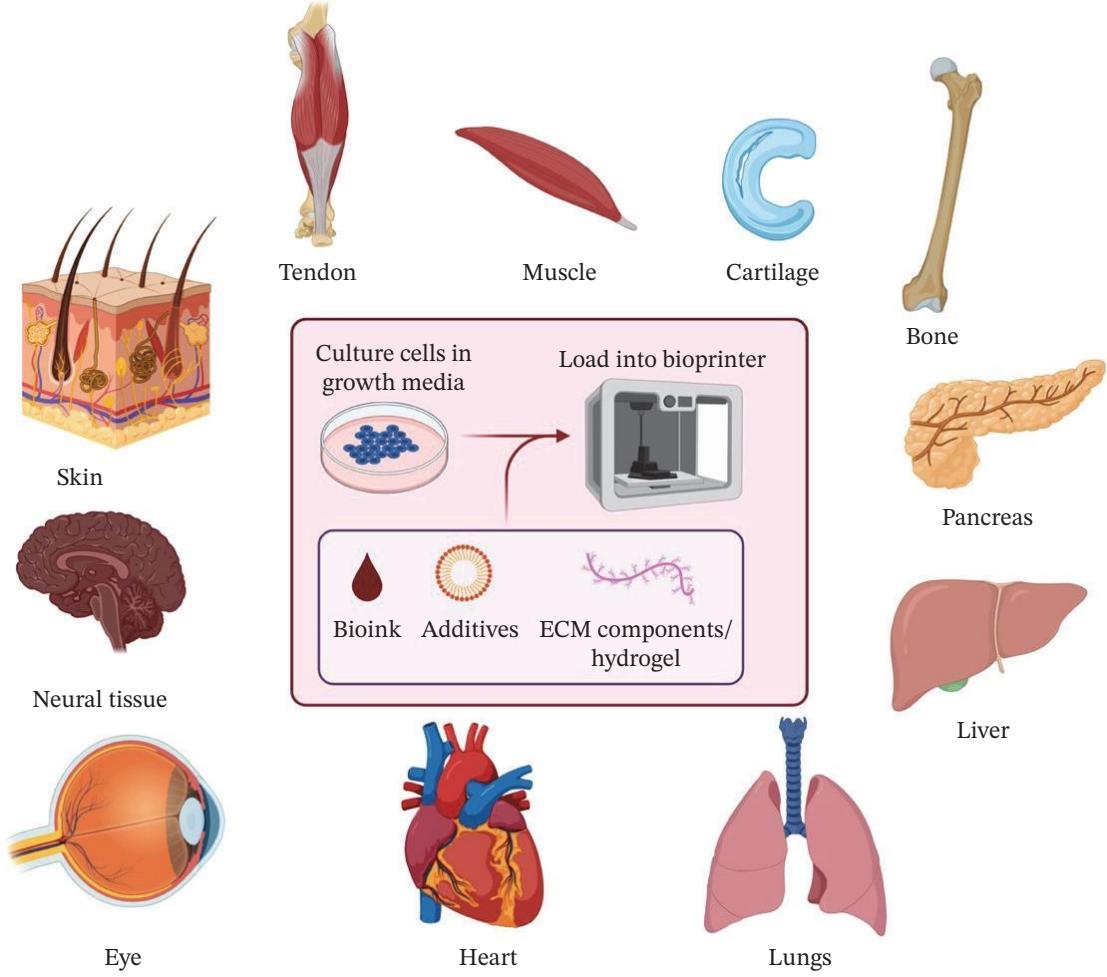
Applications of 3D bioprinting in the regeneration of body tissues. It should be noted that the majority of the tissue constructs depicted are based on preclinical animal model studies, and most applications shown remain at the experimental stage. Clinical translation in humans has not yet been achieved for most of these organ systems.

While the number of 3D‐printed implants entering clinical trials remains limited, emerging bioprinting methods that harness stem cells and native inks aim to mimic tissue complexity. These efforts target functional implantable constructs or in vitro disease models. The architecture and function of implants are closely tied to tissue physiology, thereby imposing specific constraints. Current clinical approaches prioritize restoring damaged tissue function, yet several limitations highlight the need for alternative tools. This section explores bioprinted tissues using stem cells, showcasing studies that have successfully generated physiological tissues and contributing significantly to the advancement of clinical therapies for diseased or damaged tissues (Figure 3).

### 6.1. Skin

Skin, the largest organ, is highly vulnerable to deep injuries such as burns, which can lead to scarring and the loss of hair follicles and sweat glands, thereby affecting the quality of life [186]. Autologous skin transplantation, a common treatment, faces donor shortages, secondary injuries, and infection risks [187].

One study developed ADSC‐laden bioactive scaffolds using GelMA, HA methacryloyl (HAMA), and adipose‐derived extracellular matrix (adECM) bioink that combined photocrosslinking in vitro with thermosensitive crosslinking in vivo. The adECM‐GelMA‐HAMA bioink exhibited superior wettability, degradability, and cytocompatibility compared with GelMA‐HAMA alone, and it accelerated wound healing in a nude mouse model by enhancing neovascularization, collagen deposition, and tissue remodeling [188].

Another group employed a gelatin‐alginate hydrogel to bioprint gradient composite scaffolds mimicking dermal stiffness, using calcium substrates for gradient crosslinking and encapsulating ADSCs. These scaffolds demonstrated stability and biocompatibility and promoted stem cell proliferation and migration. In animal models, they enhanced angiogenesis and accelerated wound healing by improving paracrine signaling from stem cells [106].

### 6.2. Neural Tissue

Sophisticated 3D printing techniques are crucial for replicating the brain’s complexity and neural microenvironments. Despite over 50 million affected individuals worldwide, long‐term cures for central nervous system diseases remain elusive. Neurodegenerative conditions, common among the elderly, cause cognitive, memory, and motor impairments. Cell therapies offer promise for treating conditions like Alzheimer’s, Huntington’s, Parkinson’s, ALS, stroke, and spinal cord injury [189, 190].

Using droplet printing, researchers fabricated layered cerebral cortex‐like tissues by differentiating human iPSCs into upper‐ and deep‐layer neural progenitors and printing them with a Matrigel bioink. These constructs expressed layer‐specific biomarkers and integrated structurally and functionally upon implantation into ex vivo mouse brain explants, as evidenced by neuron migration and synchronized Ca^2+^ oscillations across tissue interfaces [191].

Additionally, extrusion bioprinting assembled cortical neurons and glial cells in fibrinogen‐thrombin bioinks optimized for cell survival and printability, with HA enhancing maturation and synaptic connectivity. The printed neural tissues supported functional neural networks, including glutamatergic and GABAergic synapses, and astrocyte incorporation further improved the tissue functionality. These models effectively recapitulated disease phenotypes such as Alexander disease, demonstrating altered synaptic density and calcium signaling [192].

### 6.3. Eye

The cornea, an avascular, transparent tissue crucial for focusing light onto the retina, is supported by the corneal endothelium (CE) layer, which regulates hydration [193]. Corneal endothelial dysfunction often requires corneal transplantation (CT), but donor shortages are a major challenge [194].

A novel multi‐material EBB method was developed to recreate the human corneal stroma using hADSCs and HA‐based bioinks of varied stiffness. Alternating perpendicular layers of acellular, stiff, and cell‐laden softer HA bioinks supported mechanical integrity and guided cellular organization. The constructs exhibited high cell viability (>98%), robust proliferation, and enhanced connexin 43 expression, indicating functional cell–cell communication. Integration in an ex vivo porcine cornea model demonstrated strong attachment without additional support, highlighting the potential of this strategy to generate organized, heterogeneous microstructures suitable for corneal tissue engineering [195].

### 6.4. Lung

Lung tissue engineering (LTE) aims to produce functional lung tissues, improve vascularization, and ensure long‐term functionality to address conditions such as COPD, pulmonary fibrosis, and lung cancer [196, 197].

A recent study developed a novel bioink composed of regenerated SF and TEMPO‐oxidized bacterial cellulose (OBC) nanofibrils for extrusion‐based 3D printing. The SF‐OBC ink enabled the fabrication of multi‐layered scaffolds with enhanced mechanical properties, guided lung epithelial stem cell (LESC) alignment and proliferation, supported differentiation, and mimicked ECM remodeling, while its rapid degradability reduced the risk of foreign body reactions [109].

### 6.5. Heart

Myocardial infarction remains the leading cause of cardiovascular disease‐related deaths, accounting for 80% of cases, and often requires heart transplantation. The limited availability of donor hearts and the immunological risk of rejection have motivated the development of bioprinted cardiac constructs derived from patient‐specific iPSCs. Several studies have explored innovative biomaterials and bioprinting techniques to fabricate such constructs.

One study developed a composite hydrogel of gallic acid‐functionalized HA, collagen I, and HA‐coated multiwalled carbon nanotubes (MWCNT), which exhibited shear‐thinning behavior, reduced matrix shrinkage (~20% vs. >90% in collagen I), and supported hiPSC‐derived cardiomyocyte proliferation and function in printed constructs [198]. Another approach utilized GelMA‐xanthan gum (XG) hydrogels with improved mechanical strength (~9 kPa), printability (0.98), and cardiomyocyte differentiation from hiPSCs, with spontaneous contractions observed within 8 days and the expression of cardiac‐specific markers [199].

Furthermore, a conductive and mechanically robust bioink, GelGOMA‐gelatin methacryloyl, covalently linked to graphene oxide, enabled the printing of stress‐bearing cardiac constructs, including a functional fish heart model. GelGOMA supported the contraction and maturation of hiPSC‐derived cardiomyocytes, indicating its utility in bioelectronic and cardiac applications [200]. Additionally, scaffold‐free, tubular‐engineered heart tissues (T‐EHTs) bioprinted from hiPSC‐derived cardiac organoids exhibited enhanced vascularization, striation, and maturation in vivo when implanted in NOG mice, highlighting their potential as beating grafts for congenital heart defects [201].

### 6.6. Liver

The liver presents one of the most demanding targets for bioprinting because its function depends on the precise spatial organization of at least six distinct cell types across a repeating lobular unit ~1 mm in diameter. Reproducing this architecture requires both high‐resolution patterning and a bioink that supports the oxidative metabolism of primary hepatocytes [202, 203].

Recent work using hepatocyte organoids (HOs) derived from human chemically induced pluripotent stem cells (hCiPSCs) demonstrated improved viability and maturation via microwell devices that enhanced oxygenation. When bioprinted into 3D hepatic tissue constructs (3DP‐HOs) and implanted in mice with liver failure, these tissues promoted survival, reduced injury and inflammation, and supported liver regeneration [204].

Microarray 3D bioprinting enabled the generation of uniform human liver organoids (HLOs) from iPSC‐derived progenitors using droplet‐based bioprinting and Matrigel. These HLOs maintained high viability, robust albumin secretion, and CYP3A4 activity and offered a scalable platform for hepatotoxicity testing [205]. To further enhance scalability and reproducibility, researchers developed standardized protocols for HLO production using microarray 3D bioprinting on pillar plates. This platform enabled the uniform seeding of iPSC‐derived foregut cells, resulting in assay‐ready organoids suitable for compound screening. The integration of stem cell differentiation and bioprinting technologies improved throughput and functional relevance for high‐throughput screening (HTS) applications [206].

To address the limitations of endoderm‐only HLOs, vascularized liver organoids (vHLOs) were developed using iPSC‐derived endodermal and vascular progenitor cells. These bioprinted vHLOs showed superior hepatic functions, including albumin secretion, bile acid transport, and coagulation factor production. When expanded on pillar plates as eHLOs, they displayed enhanced nutrient diffusion and maturity, supporting advanced modeling and screening applications [207].

Additionally, 3D extrusion bioprinting using an alginate‐gelatin bioink supported the formation of biomimetic hepatic tissue from hiPSC‐derived hepatocytes, promoting spheroid formation, viability, and functional protein expression. Upon exposure to acetaminophen, the constructs demonstrated a relevant hepatotoxic response, highlighting their potential for in vitro drug testing [82].

### 6.7. Pancreas

The pancreas, housing various cell types such as *α, β, δ, γ*, and *ε* cells within a vascularized 3D structure, produces crucial hormones that regulate blood glucose levels and appetite [208]. In conditions such as type 1 diabetes, where β‐cell loss occurs, transplantation of these cells or pancreatic tissue is necessary, but challenges such as donor shortages persist [209].

A recent study employed EBB to fabricate pancreatic constructs that integrate endocrine and exocrine compartments using mouse pluripotent stem cell‐derived pancreatic progenitors, pancreatic endothelial cells, and MSCs. Co‐printing endothelial cells and MSCs formed vasculature‐like networks with high cell viability and organization. Although progenitor‐laden fibrin constructs showed differentiation, their structural integrity declined over time. Incorporating an alginate base layer improved shape retention and endocrine differentiation. Additionally, alginate‐fibrin bioinks combined with kappa‐carrageenan (CarGrow) support baths enhanced the structural stability and cellular differentiation of both pancreatic and endothelial cells [210].

### 6.8. Bone

Bone tissue plays a critical role in organ protection, structural support, locomotion, and mineral homeostasis. Its development during embryogenesis is orchestrated by ossification, which involves the condensation and differentiation of BMSCs into osteoblasts or chondrocytes, ultimately forming mineralized bone tissue [211]. Although autologous grafts remain the clinical gold standard for skeletal defect repair, their limitations, including donor‐site morbidity, necrosis due to poor vascularization, and inadequate integration with the host tissue, have prompted exploration of stem cell‐based strategies combined with 3D bioprinting technologies [212]. Table 2 summarizes the various types of stem cells and scaffolds used in 3D bioprinting for bone tissue engineering applications.

**Table 2:** Overview of stem cell types utilized in 3D bioprinted scaffolds for bone tissue engineering applications.

Bioink	Cell types	Main findings	Refs.
Acylated gelatin bioink with Dex‐M and BMP2	hMSCs	Dex‐M+BMP2+G‐scaffold significantly enhances osteogenic differentiation and mineralization of hMSCs	[213]
PCL, co‐printed with GELMA	hBMSCs	3D GELMA‐based biomimetic periosteum with a 1:1 ratio of MSCs to osteoblast enhanced osteogenic differentiation and matrix mineralization	[214]
Pluronic F127 and custom‐synthesized poly(2‐methyl‐2‐oxazoline)‐block‐poly(2‐n‐propyl‐2‐oxazine) (POx/POzi)	hMSCs	Showed that POx/POzi hydrogel supported short‐term hMSCs‐TERT viability with minimal stress response	[215]
Methacrylate modified Agarose	MSC	The bioink enhanced printability, supported MSCs viability, and promoted osteogenic differentiation	[216]
GELMA and polyethylene glycol‐modified barium titanate nanoparticles	BMSCs	The piezoelectric hydrogel scaffold with stem cells enhanced bone regeneration and accelerated defect healing by supporting cell growth and osteogenic activity	[217]
Methacrylated alginate/gelatin‐methylcellulose (AGM)	BMSCs	The scaffold promoted BMSC proliferation, osteogenic differentiation, and neoangiogenesis	[218]
Methacrylated hyaluronic acid (MEHA)	hMSCs	Embedded bioprinting with MEHA hydrogels enhanced with bone allograft or TCP particles created dense, osteoinductive constructs, promoting osteogenic differentiation of hMSCs without external growth factors	[219]
Hyaluronic acid methacrylate (HAMA)	Human periosteum‐derived cells (hPDCs) and hBMSCs	hPDC spheroids in a HAMA matrix showed superior mineralization and hydrogel colonization compared to hMSCs spheroids	[220]
Nanohydroxyapatite (NHA)	hBMSCs	Mg^2+^ and CO_3_^2−^ doped NHA nanoparticles improved bioink printability and boosted the viability, metabolic activity, and osteogenic differentiation of hBMSCs	[221]
Alginate‐gelatin	hBMSCs	Alginate‐gelatin‐based bioinks enhanced with CNFs, NHA, and RGD‐alginate improved printability, supported hMSCs viability, and promoted osteogenic differentiation	[222]
Gelatin methacryloyl bone matrix anhydride (GBMA)	BMSCs	The 3D bioprinted GBMA@ BMSCs hydrogel with Hif1a activation accelerated fracture healing by improving cartilage callus formation and regenerative outcomes	[223]
Gelatin and hydroxyapatite (HA)	Dental pulp stem cells (DPSCs)	Gelatin‐nano HA scaffolds with over 60% porosity induced osteogenic differentiation in stem cells, marked by increased osteocalcin levels	[224]
Liver decellularized extracellular matrix (LDECM)	BMSCs	The BMSC‐laden LDECM‐gelatin‐alginate bioink enhanced bone regeneration by promoting osteogenesis and angiogenesis	[225]
Methacrylated bone‐derived decellularized extracellular matrix (BDECM‐MA)	BMSCs	3D‐printed BDECM‐MA combined with silicon‐substituted calcium phosphate and BMSCs demonstrated enhanced osteogenesis, angiogenesis, and immunomodulation, significantly accelerating bone defect repair	[226]
GEMLA and PCL	hiPSC‐derived mesenchymal stem cells (iMSCs)	iMSCs osteogenesis was enhanced in 3D‐printed PCL modules with a CHIR99021‐treated microenvironment, resulting in improved bone regeneration	[227]
GELMA	DPSCs	Bioprinted 10% GELMA hydrogels, when embedded with DPSCs, enhanced osteogenic differentiation and promoted cranial bone regeneration	[228]
Gelatin methacryloyl/Laponite nanoclay/N‐acryloyl glycinamide	hBMSCs	Coaxial extrusion of gelma‐Laponite‐NAGA bioink enabled stem cell loading and spatially organized dual‐cell cultures, enhancing cell viability and osteogenic differentiation	[229]
Gelatin methacrylate/alginate methacrylate/hydroxyapatite (GELMA/ALGMA/HAP)	BMSCs	GELMA/ALGMA/HAP bioink supported stem cell‐derived bone organoid maturation, advancing bone tissue engineering	[230]
Nanohydroxyapatite (nHap), and poly(ethylene glycol)diacrylate (PEGDA) with gelma	hMSCs	PEGDA‐GELMA‐based bioink significantly enhanced the biophysical properties, printability, and cytocompatibility of 3D‐bioprinted scaffolds, supporting hMSCs growth and promoting bone tissue engineering applications	[231]
Graphene oxide (GO) mixed with methyl methacrylate gelatin (gelma)	MSCs	GO‐enhanced GELMA scaffolds promoted stem cell osteogenesis and supported bone tissue engineering for defect repair	[232]
Laponite (Lap), cyp‐loaded mesoporous silica (cyp@msns) and ultrasmall superparamagnetic iron oxide nanoparticles (USPIO@SiO_2_) in GELMA	BMSCs	3D‐bioprinted composite scaffold integrating bioactive biomaterials and BMSCs significantly enhanced bone tissue engineering by enabling real‐time in situ monitoring of osteogenesis and scaffold degradation	[233]
Gelatin (GEL) and decellularized bone (DB)	hTERT‐MSCs	Minimalistic bioink composed of GEL‐DB particles, and stem cells significantly enhanced cell viability, proliferation, and osteogenic differentiation	[234]
GELMA/ALGMA	BMSCs	Anisotropic bicellular living hydrogels (ABLHS), fabricated using a bioprintable GELMA/ALGMA bioink and embedded with spatially organized stem cells, effectively promoted osteochondral regeneration by enhancing bone and cartilage tissue engineering	[235]
Zinc‐based metal‐organic framework (MOF) nanoparticles in GELMA	hASCs	The incorporation of zinc‐based MOF nanoparticles into biocompatible GELMA hydrogels significantly enhanced their bio‐printability, mechanical strength, and osteoinductive capacity by promoting the osteogenic differentiation of hASCs	[236]
Collagen and hydroxyapatite (HAP)	BMSCs	Collagen‐HAP scaffold, 3D‐printed with gelatin support, enhanced stem cell viability and osteogenesis	[118]
GELMA	Periodontal ligament stem cells (PDLSCs)	Bioprinting PDLSCs in high‐concentration GELMA hydrogels enhanced osteogenic differentiation and promoted bone tissue regeneration	[237]
GELMA, gelatin, and alginate	Rat mesenchymal stem cells (rMSCs)	3D printed dual‐crosslinked hydrogel supported stem cell viability and osteogenesis	[147]
Gelation crosslinked tyramide modified Alginate and Carboxymethyl cellulose (CMC)	hBMSCs	3D printed Alg‐Tyr‐Gel and CMC‐Tyr‐Gel crosslinked with visible light supported MSC viability and differentiation into chondrogenic and osteogenic lineages	[238]
Gelma/HAMA‐MXENE	hMSCs	MXENE‐incorporated GELMA/HAMA bioinks promoted the spontaneous osteodifferentiation of hMSCs	[239]
Gelatin, alginate, and autologous bone (AB)	BMSC	3D bioprinted AB particle scaffold, incorporating a polycaprolactone shell and BMSC‐laden hydrogel, effectively promoted bone tissue regeneration by enhancing osteogenic differentiation and recruiting native stem cells	[240]
Gelatin/silk fibroin scaffold loaded with Si_3_N_4_ nanoparticles	MSCs	3D‐printed composite scaffold made of gelatin, silk fibroin, and 1% Si_3_N_4_ effectively promoted MSCs osteogenic differentiation and bone regeneration	[241]
Alginate‐methylcellulose (ALGMC) blend functionalized with egg white (EW)	hMSCs	ALGMC + EW bioink supported high viability, adhesion, and osteogenic differentiation of stem cells	[242]
GELMA, methacrylated silk fibroin, GELDA, and graphene oxide nanosheet	BMSCs	Bioink combined with BMSCs, was successfully used to 3D bioprint an artificial periosteum that promoted strong adhesion, biocompatibility, and enhanced osteogenesis	[243]
Polypyrrole‐grafted gelatin methacryloyl (GELMA‐PPY)	hBMSCs	Triple‐cross‐linked GELMA‐PPY bioink enabled the 3D bioprinting of highly stable, conductive, and cytocompatible constructs that supported hBMSCs viability and osteogenic differentiation	[244]
GELMA	DPSCs	Low‐concentration GELMA served as an effective biomaterial for bioprinting ephrinb2‐overexpressing DPSCs which maintained high viability and significantly enhanced osteogenic differentiation	[245]
Fibrin based bioinks and bioprinted into PCL frameworks	hMSCs	Fibrin‐based bioinks with hMSC in PCL scaffolds enabled 3D bioprinting of hypertrophic cartilage templates that promoted vascularization and bone regeneration in large defects	[246]

### 6.9. Cartilage

Cartilage has limited regenerative capacity, often necessitating prolonged healing and treatments such as prosthetics or grafts for degenerative osteoarthritis [247]. Current treatment involves autologous cartilage implantation, harvesting tissues from the patient for implantation [248]. Table 3 summarizes the various types of stem cells and scaffolds used in 3D bioprinting for cartilage tissue engineering applications.

**Table 3:** Overview of stem cell types utilized in 3D bioprinted scaffolds for cartilage tissue engineering applications.

Bioink	Cell types	Main findings	Refs.
Fibrin, nanocellulose and HA	Embryonic‐derived mesenchymal stem cells (ES‐MSCs)	Fibrin‐based bioinks with ES‐MSCs enabled in situ bioprinting and supported cartilage tissue engineering in osteoarthritic defects	[249]
Glycyrrhizic acid/methacrylate‐acylated hyaluronic acid (GA/HA/G‐Exos)	Synovial mesenchymal stem cells (SMSCs)	GA/HA scaffolds loaded with GDF‐5‐preconditioned SMSC‐derived exosomes enhanced cartilage regeneration, highlighting a stem cell‐free biomaterial strategy for cartilage tissue engineering	[250]
Water‐soluble acellular cartilage matrix (ACM) + HA and GELMA	hADSCs	3D‐printed photocrosslinkable acellular cartilage matrix and gelatin‐based biomaterials with co‐cultured stem cells and chondrocytes improved regeneration and mechanics of tissue‐engineered auricles by integrating perichondrium and cartilage	[251]
Dex‐loaded poly(ethylene glycol) diacrylate (PEGDA) guest polymers with acryloyl β‐cyclodextrin (AβCD) host monomers, in combination with cobalt nanowires (Co NWs)	Umbilical cord‐derived mesenchymal stem cells (UMSCs)	3D bioprintable hypoxia‐mimicking hydrogel supported UMSCs to promote cartilage regeneration and immunomodulation, offering a promising biomaterial for osteoarthritis treatment	[252]
GelXA cartilage bioink	hADSCs	Dual (photo + ionic) crosslinking of hydrogels provided optimal mechanical strength and high stem cell viability, highlighting its potential as a standardized biomaterial for cartilage tissue engineering applications	[253]
Alginate/gelatin/chondroitin sulfate and graphene oxide	MSCs	3D‐printed biomimetic hydrogel significantly enhanced MSC viability and chondrogenic differentiation	[254]
GELMA and chitosan methacrylate (CHMA)	BMSCs	3D printed photo‐crosslinkable gelatin‐chitosan hydrogel promoted stem cell aggregation and cartilage formation, offering a promising biomaterial for cartilage tissue engineering	[255]
Alginate, gelatin, and fibrinogen	MSCs	Type I collagen concentration in the bioink guided stem cell‐driven cartilage tissue engineering by enabling layer‐specific hyaline cartilage formation	[256]
Methacryloyl‐modified acellular Wharton’s jelly (AWJMA) and GELMA	BMSCs	AWJMA and GELMA bioink, served as an effective biomaterial for 3D bioprinting, supporting BMSC viability and chondrogenic differentiation, and thereby significantly advanced cartilage tissue engineering for full‐thickness articular cartilage defect repair	[257]
Ac‐Ile‐Ile‐Cha‐Lys‐NH2 (IIZK) and Ac‐Ile‐Cha‐Cha‐Lys‐NH2 (IZZK)	hBMSCs	Ultrashort peptide bioinks supported stem cell chondrogenesis and enabled zone‐specific cartilage bioprinting	[258]
Gelatin/Hyaluronic acid (HyA)	hMSCs	Adjusting the mechanical properties of gelatin/HyA 3D scaffolds influenced stem cell differentiation	[259]
GELMA	MSCs	GelMA‐MSCs scaffold, fabricated by 3D bioprinting, effectively supported stem cell viability and promoted cartilage tissue regeneration, resulting in significant cartilage repair in a rabbit injury model	[260]
Xanthan Gum‐Alginate	hMSC	Bioprinting hMSC spheroids in a xanthan gum‐alginate hydrogel produced stable cartilage constructs with high cell viability and matrix production	[261]
GELMA and silk fibroin‐gelatin (SF‐G)	BMSCs	3D bioprinted silk fibroin‐gelatin constructs enhanced stem cell growth and cartilage regeneration better than gelatin methacryloyl constructs	[262]
Photo‐cross‐linked ECM	SMSCs	Photo‐cross‐linked ECM bioink supported stem cell‐driven cartilage regeneration and functional joint repair	[263]
Hyaluronic acid and MWCNT	hMSC‐AT	Combining hyaluronic acid, MWCNT, and 2‐phospho‐L‐ascorbic acid improved stem cell viability in 3D bioprinted cartilage tissue engineering constructs	[264]
Tyramine‐functionalized hyaluronan (HAT) and alginate	hBMSCs	Smart hydrogel system enabled 4D bioprinting of curved scaffolds supporting hBMSCs viability and cartilage‐like tissue formation	[265]
Alginate‐methylcellulose (algMC) bioink with collagen‐based artificial extracellular matrix (aECM)	hTERT‐MSC	Collagen‐based matrix with alginate‐methylcellulose and tannic acid improved stem cell adhesion and cartilage tissue engineering	[266]

### 6.10. Muscle

Volumetric muscle loss (VML) from trauma, tumor resection, or advanced neuromuscular disease cannot be repaired by current surgical techniques. Bioprinted constructs incorporating aligned myogenic progenitors are the leading strategy for restoring contractile function at scale [267, 268].

One approach involved a novel hybrid bioink (GH‐ASG) comprising silanized acrylic graphene oxide nanosheets (APStriol@GO) crosslinked into a GelMA‐HAMA matrix. This formulation exhibited favorable physicochemical properties, ROS‐scavenging activity, and hemostatic activity, thereby enhancing rabbit ADSC viability and differentiation into smooth muscle cells (SMCs), as confirmed by SMC‐specific markers (α‐SMA and SM‐MHC). The bioink also enabled 3D bioprinting of tubular‐ and disk‐shaped constructs with improved biocompatibility and scaffold functionality [269].

Another strategy addressed VML using a magnetorheological GelMA‐based bioink with nano‐iron oxide particles and human ADSCs. Application of magnetic fields guided cell alignment, promoting mechanotransduction and upregulating myogenic genes. In vitro and in vivo studies demonstrated enhanced myotube formation and functional muscle regeneration, with nanosized particles outperforming microsized ones by activating the Wnt/β‐catenin, Hippo, and Piezo1 pathways [270].

Additionally, bioprinting with human mesenchymal progenitor cells (hMPCs) and neural stem cells (hNSCs) using an integrated tissue‐organ printer (ITOP) showed synergistic effects on skeletal muscle regeneration. Constructs made with fibrinogen‐based hydrogels and PCL scaffolds supported NMJ formation, myotube development, and superior muscle recovery in rodent models, with co‐cultures of hMPCs and hNSCs showing significantly better outcomes than hMPCs alone [271].

### 6.11. Tendon

Tendon injuries, often associated with pain, functional impairment, and a high clinical and financial burden, have continued to pose significant challenges in regenerative medicine due to tendons’ inherently limited healing capacity. This limitation stems from their low cellularity and poor vascularization. Although autologous tendon grafts remained the standard treatment, the unique biomechanical and structural properties of tendons necessitated the development of implantable materials that could more closely mimic the native tendon tissue [272].

One study bioprinted a graded tendon‐to‐bone interface (TBI) using dECM‐based bioinks derived from porcine tendon and bone, containing hASCs and bioactive factors. Tenogenic (PVA and TGF‐β) and osteogenic (HA) components supported site‐specific differentiation. The graded structure promoted fibrocartilage formation, superior TBI integration, and functional regeneration in a rabbit rotator cuff tear model [273].

Another study used 3D bioprinted PCL scaffolds functionalized with collagen and tendon stem cell‐derived exosomes (TSC‐Exos‐S) for irreparable massive rotator cuff tears. The scaffold mimicked tendon ECM and improved BMSC proliferation, migration, and tenogenic differentiation. In vitro, it supported tendon‐like tissue regeneration with mechanical outcomes comparable to autologous grafts [274].

Table 4 summarizes the recommended stem cell source, bioink properties, printing modality, conditioning strategy, and translational readiness for each tissue type discussed in this section.

**Table 4:** Tissue‐specific design recommendations for bioprinted stem cell constructs.

Target tissue/niche	Recommended stem cell source	Priority bioink properties	Preferred modalities	Conditioning strategy	Translational readiness
Skin	• ADSCs • hMSCs • Epidermal progenitors • iPSC, derived keratinocytes	• Collagen or fibrin base; 0.5–2 kPa • RGD, functionalized • Pro‐angiogenic • Rapid remodeling	• Extrusion; droplet • Coaxial for vascular channels	• Air–liquid interface culture • Angiogenic co‐culture • Perfusion for thick grafts	Moderate Bottlenecks: • Appendage regeneration • Innervation • Full‐thickness vascular integration
Cornea	• Corneal stromal stem cells • iPSC, derived corneal epithelial and endothelial cells	• Cornea‐specific dECM or collagen; 1–5 kPa • Transparent; low light scattering • Precise crosslinking required	• Extrusion • DLP • EHD	• Curved‐mold culture • Epithelial‐endothelial co‐culture • Optical quality assessment	Preclinical Bottlenecks: • Transparency retention • Curvature fidelity • Endothelial pump function
Neural tissue	• NSCs • iPSC, derived neural progenitors • Neuron‐glia co‐cultures	• 0.1–1 kPa • Laminin or IKVAV, functionalized • Low inflammatory potential • High diffusivity	• Droplet • DLP • Low‐shear extrusion	• Perfusion • Glial co‐culture • Electrophysiological validation	Low Bottlenecks: • Long‐range axonal connectivity • Vascularization • In vivo safety
Cardiac tissue	• iPSC, derived cardiomyocytes • Endothelial cells • Cardiac fibroblasts	• 8–15 kPa; electroactive (GelMA/PEDOT:PSS) • Anisotropic alignment • Fibronectin‐functionalized	• DLP • Support‐bath extrusion • Coaxial	• Electrical pacing (1–2 Hz, 1–5 V/cm) • Cyclic stretch • Perfusion bioreactor	Low Bottlenecks: • Cardiomyocyte maturation • Vascular integration • Construct scale‐up
Liver	• iPSC, derived hepatocytes • Hepatic organoids • Endothelial and stellate cell support	• Liver dECM or collagen‐HA blend; 1–5 kPa • High nutrient diffusivity • Zonation‐compatible	• Droplet • DLP • Microfluidic	• Perfusion bioreactor • Oxygen gradient control • Multicellular zonation	Moderate (in vitro); Low (implantable) Bottlenecks: • Sustained hepatic function • Host integration
Pancreas	• iPSC, derived pancreatic progenitors • Endocrine organoids • Endothelial and mesenchymal support	• Alginate or fibrin base; 0.5–2 kPa • Nutrient‐permeable • Optionally immune‐shielding	• Extrusion • Coaxial • Microfluidic	• Perfusion • Glucose‐stimulated insulin secretion testing • Endocrine‐endothelial co‐culture	Low Bottlenecks: • Beta‐cell maturation • Vascularization • Immune protection
Bone	• hMSCs • iMSCs • Periosteal stem cells	• 25–40 kPa; hydroxyapatite‐doped • PCL framework composite • Osteoinductive (BMP‐2 loaded)	• Extrusion with PCL framework • Microfluidic gradient printing	• Perfusion • Cyclic compression (5%–15% strain, 0.5–1 Hz) • LIPUS	Moderate (preclinical) Bottlenecks: • Vascularization of large constructs • Mechanical load‐bearing at scale
Cartilage/osteochondral	• hMSCs • SMSCs • Chondrogenic progenitors	• GelMA‐PCL composite; zonally graded stiffness • Hypoxia‐compatible • Slow remodeling	• Extrusion • Coaxial • 4D printing at osteochondral interface	• Dynamic compression • Hypoxic culture (2%–5% O_2_) • Osteochondral co‐culture	Moderate (preclinical) Bottlenecks: • Hypertrophic differentiation • Tidemark integration • Subchondral bone coupling
Skeletal muscle/tendon	• Myogenic progenitors • hMPCs • Tendon stem‐progenitor cells	• Aligned fibrin or collagen; 8–17 kPa • Anisotropic; fiber‐reinforced • Stiffness gradient at interface	• Extrusion • EHD for fiber alignment • Gradient printing at MTJ	• Uniaxial cyclic stretch • Neural co‐integration • Vascular support	Low to moderate Bottlenecks: • Neuromuscular junction formation • Myotendinous interface mechanics

## 7. Future Perspectives

Bioprinting of stem cell constructs has progressed from proof‐of‐concept demonstrations to preclinical models with a defined architecture and measurable function. Despite this progress, routine clinical application remains constrained by three unresolved challenges: the absence of perfusable vascular networks in constructs that exceed the diffusion limit, incomplete stem cell maturation under static or short‐term culture conditions, and the lack of validated GMP manufacturing and regulatory pathways for cell‐scaffold combination products. Advancements in vascularization strategies, immune compatibility, and manufacturing science are required before clinical translation becomes feasible.

### 7.1. Overcoming the Vascularization Barrier and Other Translational Challenges

Oxygen diffusion in avascular tissue is limited to ~100–200 μm from the nearest capillary; constructs exceeding this threshold develop necrotic cores unless a perfusable vascular network is incorporated [275, 276]. Four strategies have shown experimental progress. Sacrificial printing embeds a water‐soluble template (Pluronic F127 or a carbohydrate glass lattice) within the construct, which is dissolved after crosslinking, leaving patent microchannels that are subsequently seeded with endothelial cells [277]. FRESH printing builds vascularized structures in a gelatin bath, as shown for the cardiac ventricle [278, 279]. Pericyte co‐culture exploits paracrine angiopoietin‐1 signaling and N‐cadherin‐mediated stabilization to drive spontaneous capillary formation when MSCs are printed alongside endothelial progenitors [280, 281]. Prevascularized organoid bioprinting incorporates iPSC‐derived vascular organoids containing endothelial cells, pericytes, and smooth muscle cells directly into the bioink, accelerating anastomosis with the host vasculature after implantation [57, 282]. Combining sacrificial templating with iPSC‐derived endothelial seeding offers a route to immune‐compatible vascularized constructs of clinically relevant dimensions.

Immune rejection is a separate but equally important barrier. iPSC‐derived progeny may re‐express antigens absent from the patient’s somatic cells, and residual undifferentiated iPSCs carrying pluripotency‐associated surface antigens trigger natural killer cell responses [283, 284]. CRISPR‐mediated deletion of HLA class I loci generates hypoimmunogenic “universal donor” iPSC lines, and transgenic expression of CD47 reduces macrophage‐mediated phagocytosis [285, 286]. Genomic instability in iPSC lines, including copy‐number variations and point mutations acquired during reprograming and expansion, necessitates whole‐genome sequencing of working cell banks prior to clinical use.

GMP‐compliant manufacturing of bioprinted constructs requires closed‐system bioreactors, chemically defined animal‐free media, in‐process sterility testing, and validated release criteria. Regulatory frameworks under FDA 21 CFR Part 1271 and EMA advanced therapy medicinal product guidelines are still evolving for constructs that combine living cells, scaffolds, and bioactive factors; early regulatory engagement is essential to align development programs with approvable designs [287].

### 7.2. Emerging Technologies Reshaping Bioprinting and Stem Cell Engineering

Emerging technologies are shifting stem‐cell bioprinting from construct fabrication toward integrated, controllable, and clinically translatable biofabrication systems. Rather than functioning as isolated printing methods, advanced platforms such as microfluidic/coaxial printing, EHD jetting, and 4D bioprinting are increasingly being combined with computational design, real‐time monitoring, closed‐system culture, and quality control workflows. Their translational value lies in improving vascular integration, spatial precision, dynamic tissue maturation, and reproducibility across patient‐specific constructs.

Artificial intelligence and machine learning are accelerating bioink optimization and process control. Bayesian optimization algorithms have identified optimal combinations of polymer concentration, crosslinker dose, and print speed that simultaneously maximize printability, cell viability, and mechanical properties within tens of experimental iterations, compared with hundreds required by conventional design of experiments [288, 289]. Convolutional neural networks trained on bright‐field images acquired immediately after printing predict post‐culture viability with an accuracy exceeding 90%, enabling real‐time process correction.

Single‐cell bioprinting uses acoustic force to deposit individual cells with picoliter precision, allowing the spatial reconstruction of diverse cell populations that replicate the native tissue’s cytoarchitecture [290].

Decellularized extracellular matrix (dECM) bioinks, made by solubilizing the ECM from organs or tissues, keep a tissue‐specific mix of adhesion proteins, growth factors, and glycosaminoglycans that can’t be recreated from individual parts. dECM bioinks from the heart, liver, and cartilage more effectively direct iPSC‐derived progenitors toward proper tissue lineages than GelMA or alginate [291, 292]. Together, these innovations point toward a future in which construct design is guided by quantitative mechanistic models rather than empirical intuition and fabrication is monitored and corrected in real time.

### 7.3. Decision Framework for Construct Design and Ethical Considerations

**Figure 4:**
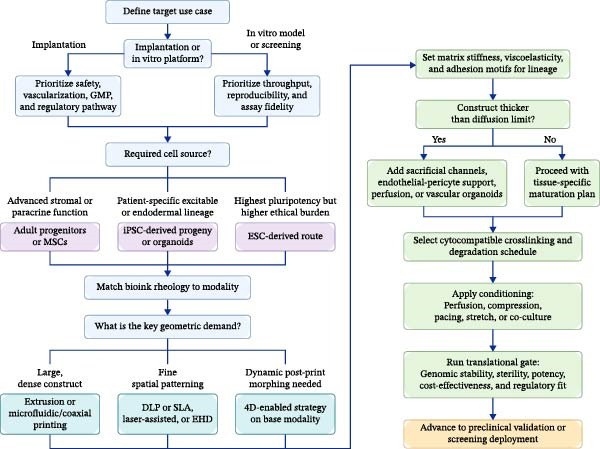
Decision framework for bioprinted stem cell construct design.

Rational construct design requires sequential decisions across target tissue definition, stem cell source selection, bioink formulation, printing modality, vascularization strategy, and post‐print conditioning. A comprehensive decision framework integrating these parameters is presented in Figure 4. Tissue‐specific recommendations for each parameter are summarized in Table 4.

Ethical considerations surrounding iPSC‐based bioprinting require careful attention. Tumorigenicity of residual undifferentiated iPSCs is a documented safety concern; current mitigation strategies include negative selection against pluripotency markers (SSEA‐4 and TRA‐1‐60) and pharmacologically activatable suicide gene systems [293, 294]. Collection and storage of somatic cells for reprograming raise questions about tissue ownership, informed consent for downstream applications, and the disclosure of genetic information to biological relatives. In resource‐limited settings, access to cGMP iPSC manufacturing infrastructure remains severely constrained, risking the inequitable distribution of advanced bioprinted therapies. Addressing this requires investment in decentralized biofabrication platforms, open‐access bioink formulations, and international harmonization of regulatory standards to facilitate technology transfer. Engagement with regulatory bodies, patient advocacy groups, and bioethics committees throughout the development process is necessary to ensure responsible clinical translation.

## Author Contributions

Concept and design: Resmi Rajalekshmi and Devendra K. Agrawal. Review of literature: Resmi Rajalekshmi. Drafting the article: Resmi Rajalekshmi. Revising and editing the manuscript: Devendra K. Agrawal. Resources and Funding: Devendra K. Agrawal. Final approval of the article: Resmi Rajalekshmi and Devendra K. Agrawal.

## Funding

Devendra K. Agrawal is supported by the R25AI179582 grant from the National Institutes of Health, USA.

## Disclosure

The contents of this research article are solely the responsibility of the authors and do not necessarily represent the official views of the National Institutes of Health. No writing assistance was utilized in the production of this manuscript. Both authors have read the manuscript and consented for its publication.

## Conflicts of Interest

The authors declare no conflicts of interest.

## Data Availability

Data sharing is not applicable to this article as no datasets were generated or analyzed during the current study.
